# Is mating failure caused by cryptic male choice in the seed bug *Lygaeus simulans*?

**DOI:** 10.1002/ece3.70341

**Published:** 2024-09-18

**Authors:** Vicki L. Balfour, Mélissa Armand, David M. Shuker

**Affiliations:** ^1^ School of Biology University of St Andrews St Andrews UK; ^2^ Department of Zoology and Evolutionary Biology, Animal Comparative Economics Laboratory University of Regensburg Regensburg Germany

**Keywords:** cryptic male choice, *Lygaeus simulans*, mate choice, mating failure, post‐copulatory sexual selection, sperm competition

## Abstract

One yet unresolved question in the study of mating system evolution is the occurrence of mating failure, when individuals go through their lives without successfully mating. This includes the failure to produce offspring even following copulation, for instance due to insemination or fertilisation failure. Copulations are costly in a variety of ways, but also a fundamental route to fitness in sexual species, and so we should expect that engaging in copulations that generate no offspring should be strongly selected against. Nonetheless, it has become apparent that mating failure is quite common in nature. Here we consider post‐copulatory sexual selection in *Lygaeus simulans* seed bugs to test the hypothesis that the high levels of mating failure found in this species (approximately 40%–60%) are caused by cryptic male choice (i.e. males choosing not to inseminate a female during copulation). In our first experiment, we found that mating failure depended on female size, but not male size, with smaller females experiencing mating failure more frequently. Mechanistically this is likely to be due to copulation duration, as shorter copulations were more likely to lead to mating failure. Likewise, copulations with smaller females were shorter. In our second and third experiments, rates of mating failure decreased when pairs were allowed to repeatedly interact with the same partner over longer durations (hours through to days), implying that mating failure is not primarily caused by infertility or chronic mechanical failure. Instead, our results strongly suggest cryptic male choice as the cause of mating failure in this species.

## Graphical abstract

We expect cryptic mating failure–the failure to produce offspring due to fertilisation or insemination failure–to be strongly selected against because copulations are costly and are also a fundamental route to fitness. Mating failure is common in nature however, and in the seed bug *Lygaeus simulans*, rates are high (40%–60%). We performed experiments to investigate the causes of mating failure in *L. simulans* and found that mating failure is not primarily caused by infertility or chronic mechanical failure, but instead evidence strongly suggests that cryptic male choice is a key driver of mating failure in this species.

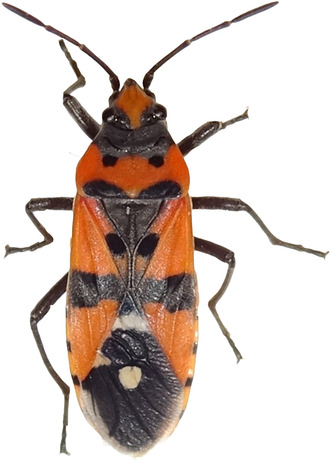

## 1 INTRODUCTION

It is becoming increasingly clear that individuals from a broad range of species often fail to successfully mate and produce offspring in their lifetimes (García‐González, 2004; Rhainds, 2010, 2019). Mating failure arises either because an individual remains a virgin until death (i.e. fails to successfully find a mate) or because copulation events fail to result in the production of offspring due to insemination and/or fertilisation failure. This latter form of mating failure has been termed cryptic mating failure (Greenway et al., 2015). Throughout this paper, when we refer to ‘mating failure’ we will be referring to this latter, ‘cryptic’ mating failure. Cryptic mating failure remains something of an evolutionary puzzle, as at first glance mating failure should be opposed by both natural and sexual selection on primary and secondary sexual function. Cryptic mating failure has a number of possible causes: for example, if one or both individuals in the copulating pair are sterile (García‐González, 2004); if there is temporary infertility, for example due to disease, malnutrition, abnormalities in sperm, or low sperm count (Sheldon, 1994); if polyspermy occurs (Frank, 2000; Greenway et al., 2015); if there is mechanical failure in terms of the incorrect coupling of genitalia (García‐González, 2004; Greenway et al., 2015); or cryptic female choice (Eberhard, 1996; García‐González, 2004; Greenway et al., 2015).

Post‐copulatory sexual selection—selection which occurs during or after copulation—is typically thought to be driven by one of two processes: sperm competition and cryptic female choice (Birkhead & Pizzari, 2002; Eberhard, 1996; Parker, 1970, 1998; Simmons, 2001; Smith, 1984; Wigby & Chapman, 2004). Cryptic female choice (CFC) occurs when females bias or skew paternity towards favoured males (i.e. fertilisation success is non‐random with respect to male phenotype) through unseen processes within the female reproductive tract, hence the term ‘cryptic’ (Arnqvist, 2014; Birkhead & Pizzari, 2002; Eberhard, 1996; Firman et al., 2017). These processes can include the expulsion of sperm from disfavoured males (e.g. in feral fowl *Gallus gallus domesticus*: Pizzari & Birkhead, 2000), or by influencing copulation duration and prematurely ending copulations with less preferred males (e.g. in the guppy *Poecilia reticulata*: Pilastro et al., 2004, 2007). Moreover, females may be able to affect the swimming speed of sperm (e.g. in ocellated wrasse *Symphodus ocellatus*: Alonzo et al., 2016). This list is by no means exhaustive (see Firman et al., 2017 for more examples). In recent decades, however, it has become apparent that cryptic male choice (CMC) also occurs within the animal kingdom (Arnqvist, 2014; Aumont & Shuker, 2018; Bonduriansky, 2001).

As with CFC, CMC occurs during or after copulation and enables males to bias their reproductive investment towards females with favoured traits, hence shaping female post‐copulatory reproductive success (Arnqvist, 2014; Aumont & Shuker, 2018; Bonduriansky, 2001). This can include transferring differential amounts of resources, such as ejaculates and nuptial gifts, to females, depending on the quality of that female (i.e. male reproductive investment is non‐random with respect to female phenotype). Strategic sperm allocation by males can therefore occur in two contexts. First, in terms of sperm competition, whereby males may strategically allocate sperm depending on the perceived risk of sperm competition, such as presence/absence of rival males (Kelly & Jennions, 2011). Second, in terms of male mate choice (Arnqvist, 2014; Aumont & Shuker, 2018; Bonduriansky, 2001).

Current evidence for cryptic male choice is limited, although there are examples. For instance, males may transfer larger ejaculates to higher‐quality females, as in the Indian meal moth, *Plodia interpunctella*, where males transfer a greater number of sperm to heavier and more fecund females (Gage, 1998). Male *Drosophila melanogaster* also vary the amount of sperm transferred to females, depending on female size, mating status, and age (Lüpold et al., 2011), although separating out sperm allocation with respect to sperm competition versus female quality (and indeed, active female cryptic choice) is difficult. Another form of CMC is that males may also copulate for longer with preferred females, such as in the soldier fly *Merosargus cingulatus*, in which males spend more time in copula with larger females (Barbosa, 2011). Finally, one yet unproven hypothesis is the idea that males may choose to withhold entire ejaculates during copulation with unfavoured females. This may be beneficial if prolonged mate assessment, available during the preliminary stages of copulation prior to insemination, makes rejecting a female and not investing any sperm in that female's eggs the optimal strategy for a male.

Here we consider whether the considerable extent of mating failure seen in the seed bug *Lygaeus simulans* is due to cryptic male choice for larger females. In this species, approximately 40%–60% of copulations fail to result in offspring production (Balfour, Corliss, et al., 2024; Balfour et al., 2020; Greenway et al., 2017; Greenway & Shuker, 2015; Micholitsch et al., 2000; Tadler, 1999; Tadler et al., 1999). This high occurrence of mating failure appears to be due to a lack of sperm transfer, as over 90% of females which failed to produce offspring after a copulation event had no sperm present in their spermatheca (Greenway et al., 2017). Both females and males mate multiply in this species, and it is thought that this might be a means of mitigating the costs of mating failure (Shuker et al., 2006), as bugs which copulate more frequently are more likely to successfully produce offspring (Balfour et al., 2020). In terms of male mate choice, there is often pre‐copulatory mate choice for larger females, as copulations are far more likely to occur when pairings involve large females (Balfour et al., 2020). There is some evidence that larger males are also more likely to engage in copulation, but the difference in mean body length between males that copulate and those that do not is much smaller than the difference in female body length (Balfour et al., 2020; Dougherty et al., 2015; Dougherty & Shuker, 2016). Whether these patterns are due to larger individuals being preferred due to mate choice, or whether larger individuals are just more willing to mate, is as yet unclear. In terms of post‐copulatory processes, there is evidence that copulations involving larger females last for longer and are less likely to result in mating failure (i.e. smaller females are more likely not to receive sperm: Dougherty & Shuker, 2014, 2016; Balfour et al., 2020). Males may perceive these females to be of higher quality, as larger females are expected to be more fecund, lay more eggs, and therefore have a higher potential for producing a greater number of offspring. Males would therefore be expected to copulate with larger females for longer to either (a) transfer sperm, (b) transfer a greater number of sperm, (c) guard the female for a longer time against copulations from rival males, or (d) a combination of all three.

We performed three experiments to try and untangle why so many copulations fail to generate offspring in *L. simulans*. We first asked whether cryptic mating failure is caused in part by CMC. In Experiment 1, we separated males and females into different size classes and paired up individuals in different size combinations and looked at both pre‐ and post‐copulatory selection. If cryptic mating failure is caused by cryptic male choice for higher quality (i.e. large) females, then we would predict that the chance of mating failure should depend on female but not male size and that males should be more likely to inseminate larger females.

We also wanted to check whether mechanical failure and/or high levels of genetic incompatibility could explain the patterns of cryptic mating failure we see, as an alternative to CMC. One way to test this is to pair individuals for longer, to allow (or encourage) repeated matings. We thus paired up males and females for different set periods of time (Experiment 2: 1–24 h; Experiment 3: 1–5 days) and then assessed whether mating failure occurred and, if it did not, how many offspring pairs produced. We hypothesised that longer copulations, or multiple copulations, increase the amount of time for sperm transfer and so decrease the chance of mating failure. From this we predicted that the chance of mating failure would decrease the greater number of hours/days bugs were housed in pairs.

## 2 MATERIALS AND METHODS

### 2.1 Ethics statement

All work carried out complied with local and national animal welfare regulations. Our work involved the use of insects (*Lygaeus simulans*) for which no review is necessary for carrying out experiments. There are no welfare or environmental implications of the experimental design or procedures.

### 2.2 Husbandry

Wild‐type *Lygaeus simulans* were collected in Tuscany, Italy, in 2008 and 2009. Bugs were maintained in continuous culture population boxes (30 × 15 × 15 cm plastic boxes) and provided with ~500 g of de‐husked organic sunflower seeds, 2–3 cotton‐bunged tubes of deionised water (25 mL) and cotton wool for habitat. Water tubes were changed weekly. Population boxes were kept in an incubator at 29°C on a 22:2 h light:dark cycle to prevent the onset of reproductive diapause. New population boxes were created approximately every 6–10 weeks by transferring around 50 bugs of each instar category to a fresh box using an aspirator. Bugs were always taken and mixed from at least two different population boxes to limit inbreeding depression.

To obtain virgin adults for experiments, we transferred late instar nymphs to nymph boxes (20 × 10 × 8 cm plastic boxes) and provided them with an ad libitum supply of sunflower seeds, a cotton‐bunged tube of deionised water (25 mL) which was changed weekly, and cotton wool for habitat. Nymph boxes were checked every 2–3 days for newly eclosed adults to ensure virginity (achieving reproductive maturity takes 5–6 days: Burdfield‐Steel et al., 2015). Adults were transferred to collection tubs (108 × 82 × 55 mm plastic deli tubs) using soft forceps and were separated by sex and phenotype. A maximum of 10 individuals were housed together in one collection tub and were provided with an ad libitum supply of sunflower seeds, a cotton‐bunged tube of deionised water (7 mL), and a piece of cotton wool for habitat. All boxes and tubs were kept in the incubator.

### 2.3 Experiment 1: pre‐ and post‐copulatory sexual selection for body length

For this experiment we wished to maximise male and female size variation across treatments. Wild‐type *L. simulans* were pre‐measured 24–48 h prior to the experimental trial. Bugs were classified as either large (L), medium (M), or small (S). Bugs in the medium category were the mean length of wild‐type bugs ± half a standard deviation (SD). The means and SDs of males and females were taken from Balfour et al. (2018). Exact sizes for each of the categories are shown in Table 1. We (VLB) measured the bugs by placing them between two glass slides and pressing gently to hold the bug still, but not so hard that any damage was done to the bug. The bug was then placed under a calibrated dissecting microscope fitted with an eyepiece micrometre, and the body length from the end of the snout to the tip of the wings was measured. For this experiment, we used only males and females which were either large (L) or small (S). Bugs were then kept in tubs (108 × 82 × 55 mm plastic deli tubs) of up to 10 individuals of the same sex and size category and provided with cotton wool, a cotton‐bunged water tube (7 mL) and an ad libitum supply of sunflower seeds. Bugs were returned to the incubator until the start of the experimental trial.

**TABLE 1:** Size categories for male and female wild‐type *Lygaeus simulans* based on the mean size of *L. simulans* bugs used in Balfour et al. (2018).

Sex	Size category	Body length (mm)
Female	Large	>11.8
	Medium	11.4–11.8
	Small	<11.4
Male	Large	>10.9
	Medium	10.5–10.9
	Small	<10.5

*Note*: The medium category was calculated as the mean ± half the standard deviation (SD). The large category was above this range and the small category below.

**FIGURE 1:**
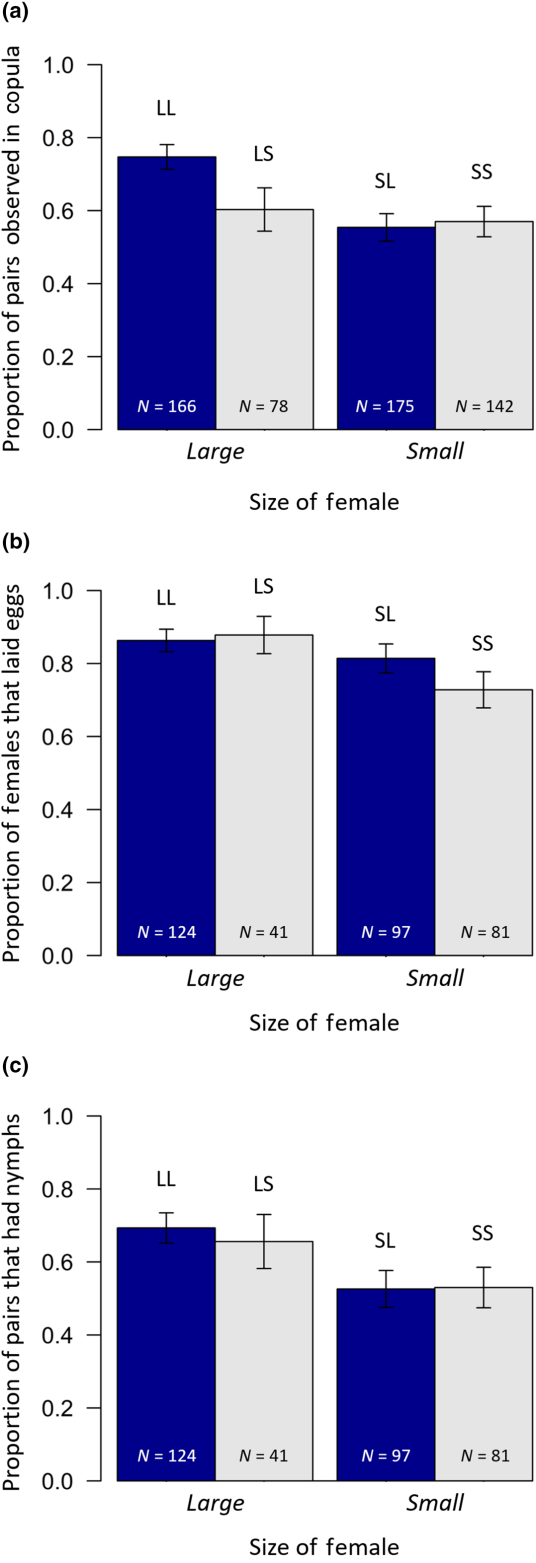
The proportion of (a) pairs observed in copula, (b) females observed in copula which subsequently laid eggs and (c) pairs which were observed in copula and successfully produced nymphs, depending on female size, in Experiment 1. Solid blue bars represent pairs in which the male was large, grey bars the pairs in which the male was small. Error bars represent the standard error. Treatment codes are shown above each bar (first letter = female size; second letter = male size; L = large; S = small), and sample sizes are given on each bar.

We had four experimental combinations using the large and small males and females (four treatments: LL, LS, SL, SS; the first letter denoting size of female, and second letter denoting size of male, L = large, S = small). On day 1, we paired up virgin males and females (7–15 days post‐eclosion) pseudo‐randomly (i.e. for treatment LS, we randomly selected a large female and paired her up with a randomly selected small male) in 55 mm diameter Petri dishes and allowed them to mate for 6 h. During the 6 h trial we observed pairs every 15 min and recorded whether they were engaged in the classic back‐to‐back copulation position (yes/no). If pairs formed the back‐to‐back position but broke apart after two consecutive checks or less (<30 min), then they were left to re‐mate. This is because the shortest period of time required for successful insemination is 30 min (Gschwentner & Tadler, 2000). We emphasise that for all analyses throughout this paper, end‐to‐end pairings <30 min in length were not counted as copulations. This is not to suggest that these short male–female interactions may not be important, but here we wish to focus primarily on copulatory pairings where there is the *potential* for sperm transfer to take place, and then look to see if it actually does take place, assayed by the production of fertilised eggs and hence offspring (see below). If pairs broke apart after copulating for three consecutive checks or more (>30 min), then we recorded this as a copulation, recorded the duration, and then separated the pair, euthanising the male by placing it in the freezer at −18°C, and placed the female in an individual tub (108 × 82 × 55 mm plastic deli tub) with a cotton‐bunged water tube (7 mL) and 20–30 sunflower seeds. At the end of the 6 h trial all remaining pairs were separated, and males were euthanised while females were placed in individual tubs. If pairs were still engaged in copulation, then they were separated by gently brushing the genitalia with a paintbrush. The sample sizes across the four treatment combinations ranged from *N* = 78–175 (see Figure 1).

We allowed females to lay eggs for 7 days (note that virgin females can lay unfertilised eggs), then on day 8 we euthanised females at −18°C and checked tubs for the presence/absence of eggs, discarding any tubs without eggs. We then returned tubs to the incubator for a further week and on day 11 we froze the tubs at −18°C for a minimum of 24 h before scoring tubs for the presence/absence of nymphs and counting any nymphs present.

### 2.4 Experiment 2: how mating failure varies over 1–24 h

To investigate whether the likelihood of mating failure decreased with increasing length of time that pairs were housed together, and hence an increase in time that pairs could potentially copulate, we housed pairs together for different durations of time. Virgin pale *L. simulans* (7–13 days post‐eclosion, see Balfour et al., 2018 for a description of the pale mutant phenotype) were paired up with an individual of the opposite sex in a tub (108 × 82 × 55 mm plastic deli tub) containing a cotton‐bunged water tube (7 mL) and 20–30 sunflower seeds. We left pairs to mate unobserved in the incubator for either 1, 2, 3, 6, 12, or 24 h. The sample sizes were: *N* = 30, 30, 30, 26, 30, and 30, respectively. After the removal of data points due to the death of bugs during the paired phase, the final sample sizes were: N = 30, 30, 30, 26, 29, and 29, respectively. When the allocated time window was up, we separated pairs, removing the males and euthanising them in the freezer at −18°C. Females were allowed to lay eggs for 7 days after the pair was split up. We then also euthanised the females and checked tubs for the presence/absence of eggs, discarding any tubs without eggs. Tubs were then left in the incubator for a further 7 days before being frozen for a minimum of 24 h at −18°C. Any nymphs present in the tubs were then counted.

### 2.5 Experiment 3: how mating failure varies over 1–5 days

Experiment 3 followed the same experimental procedure as Experiment 2 with the exception that pairs were housed together for either 1, 2, 3, 4 or 5 days. The sample sizes were: *N* = 44, 34, 33, 40, and 38, respectively. After the removal of data points due to the death of bugs during the paired phase, the final sample sizes were: *N* = 43, 33, 29, 36, and 33, respectively.

### 2.6 Statistical analysis

All statistical analysis was carried out using R version 3.6.1 (R Core Team, 2019). We used a Pearson's correlation test to investigate whether there was a correlation between copulation duration and latency to mate. We used Generalised Linear Models (GLMs) with a binomial distribution and logit link function to test the effect of female size and male size and an interaction between these terms on (i) the likelihood of a pair copulating, (ii) the likelihood of a female laying eggs, (iii) the likelihood of a pair producing nymphs, and (iv) whether pairs initiated copulation within the first 15 min of the experimental trial. We also used a binomial GLM to investigate the relationship between female size, male size, and copulation duration on the likelihood of mating failure. Finally, we used a Gaussian GLM with normal errors to investigate the relationship between female size, male size, and copulation duration on the number of nymphs produced. We used so‐called ‘Type II sums‐of‐squares’ throughout, with ‘*F*’ tests for the Gaussian GLMs, and likelihood‐ratio (‘LR’) tests for the binomial GLMs, presented as *χ* ^2^ test statistics. For Experiments 2 and 3 we used a binomial Generalised Linear Model with a logit link function to test the effect of length of time paired up on the likelihood of mating failure. We used a General Linear Model with a Gaussian distribution to test the effect of length of time paired on the number of nymphs produced.

## 3 RESULTS

### 3.1 Experiment 1: pre‐ and post‐copulatory sexual selection for body length

In summary, out of the 551 pairs used in the experiment, 343 of these engaged in copulation (62.3%) and 207 of these pairs produced nymphs, giving an overall mating failure rate of 39.7%. Copulation was more likely to occur if the pairing involved a large female, and these copulations lasted longer than copulations with small females and were less likely to result in mating failure. Male body length had no effect on any of these variables.

Considering the results in more detail, the likelihood of a pair engaging in copulation depended on female size (Figure 1a), with large females much more likely to engage in copulation than small females (*χ* ^2^_1_ = 10.58, *p* = .001). Though there was no influence of male size as a main effect (*χ* ^2^_1_ = 1.10, *p* = .295), there was a marginally non‐significant interaction between male and female body size on pairing success (interaction: *χ* ^2^_1_ = 3.63, *p* = .056). In Figure 1a we can see this is due to a higher proportion of pairings involving two large individuals resulting in copulation than other pairing combinations.

Of the pairs which copulated, 60.3% initiated copulation within the first 15 min of the trial, and this was independent of female size (*χ* ^2^_1_ = 1.04, *p* = .301), male size (*χ* ^2^_1_ = 0.09, *p* = .761), or any interaction between the two (interaction: *χ* ^2^_1_ = 0.64, *p* = .425). There was a significant negative correlation between copulation duration and latency to mate however (*r* = −0.53, t_341_ = −11.69, *p* < .001). This is likely because pairs which initiated copulation later had less time to mate before they were separated at the end of the experimental trial, and so this may be partly an artefact of the experimental design.

In terms of post‐copulatory sexual selection, females were more likely to lay eggs if they had copulated (81.9% versus 47.6%: *χ* ^2^_1_ = 70.52, *p* < .001). Of these females, larger females tended to be more likely to lay eggs (*χ* ^2^_1_ = 3.81, *p* = .051; Figure 1b), but there was no impact of male size (*χ* ^2^_1_ = 0.97, *p* = .324) nor an interaction between male and female size (interaction: *χ* ^2^_1_ = 0.96, *p* = .328).

The likelihood of experiencing (cryptic) mating failure (i.e. having no offspring following copulation) significantly depended on female size, with larger females being less likely to experience mating failure than small females (*χ* ^2^_1_ = 8.20, *p* = .004; Figure 1c). Male body size had no influence on mating failure (*χ* ^2^_1_ = 0.04, *p* = .839), nor again was there any interaction between male and female body size (interaction: *χ* ^2^_1_ = 0.14, *p* = .712).

**FIGURE 2:**
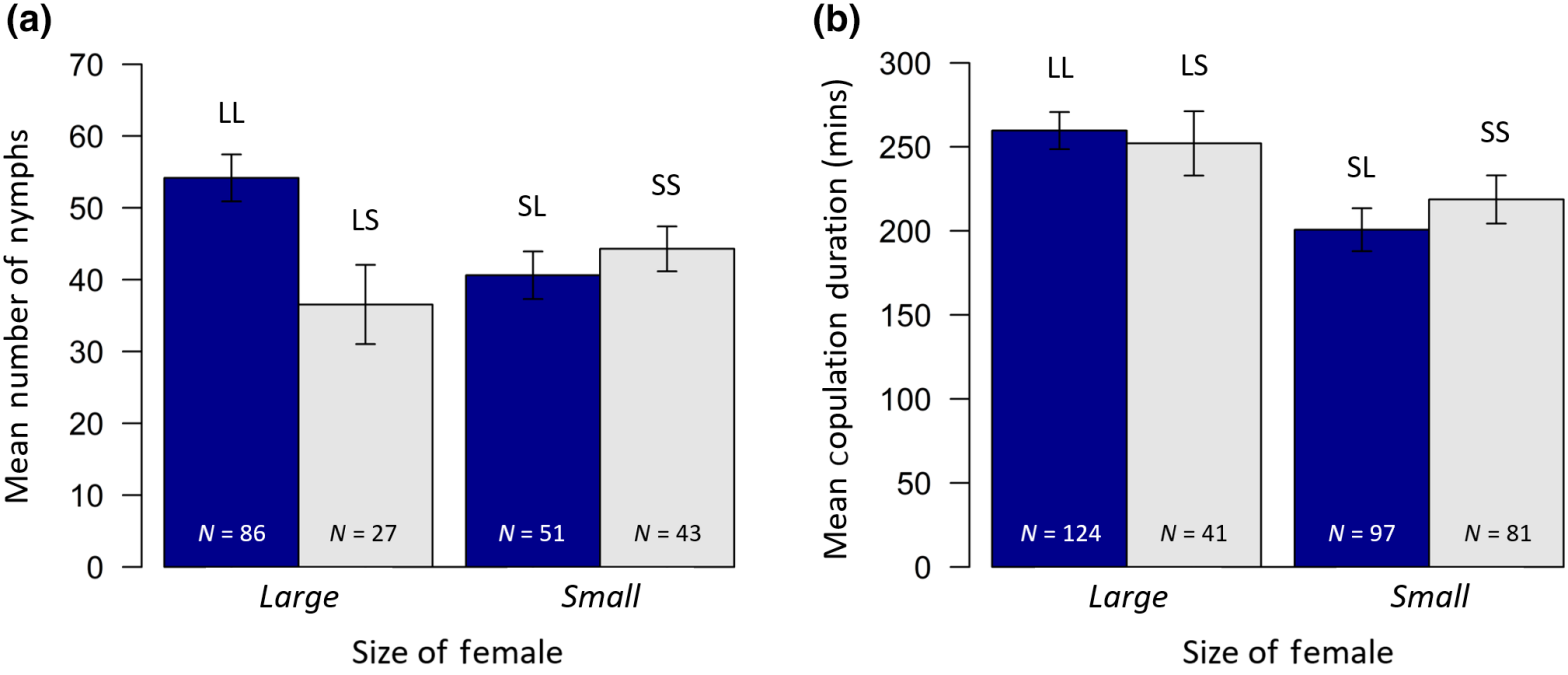
(a) Mean number of nymphs produced by pairs which had nymphs (and hence did not experience mating failure), and (b) mean copulation duration for pairs that copulated, depending on female size, in Experiment 1. Solid blue bars again represent pairs in which the male was large, grey bars the pairs in which the male was small. Error bars represent the standard error. Treatment codes are as described in Figure 1.

**FIGURE 3:**
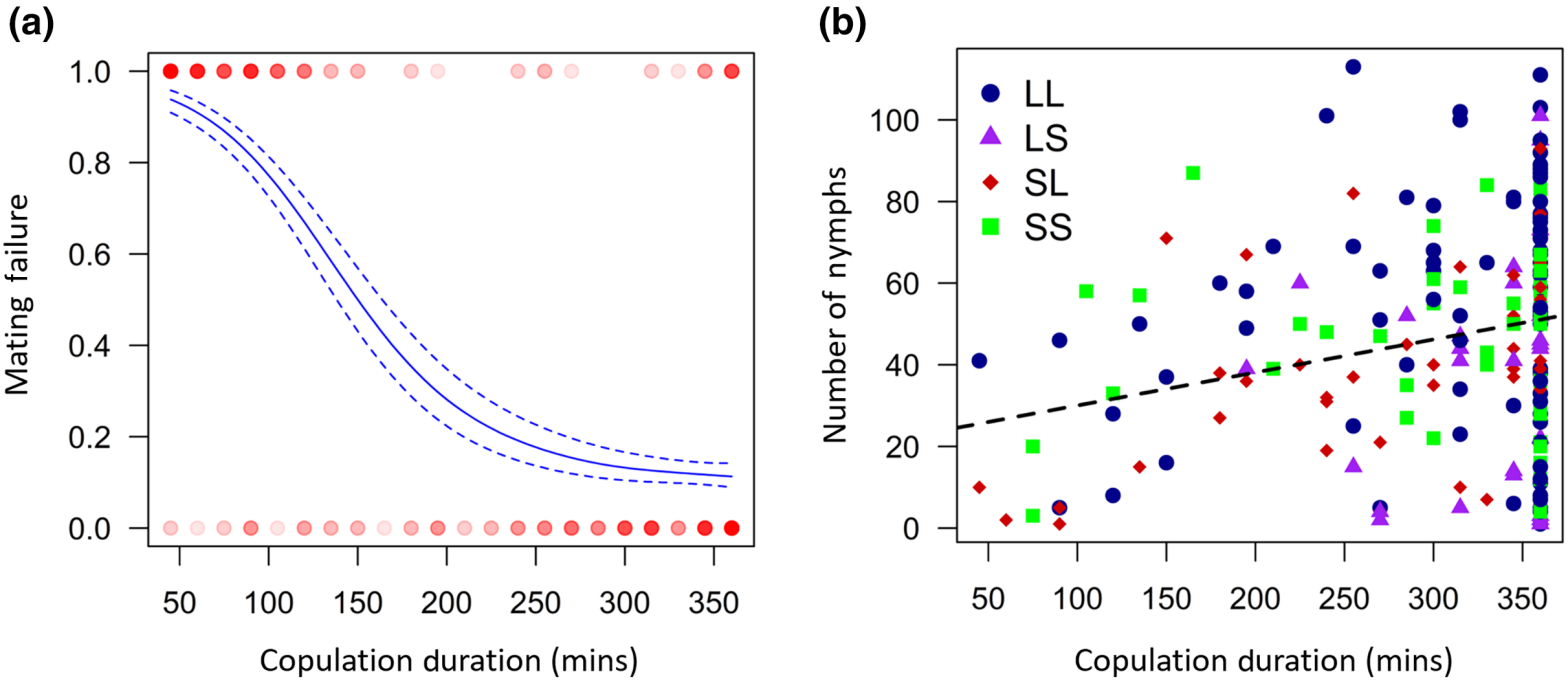
(a) Relationship between copulation duration and the rate of mating failure (whether pairs had nymphs [0] or not [1]), for pairs which mated in Experiment 1, visualised as cubic splines (*N* = 343). Data are represented by circles, with the colour reflecting the number of individuals of the given size and mated state (darker = more replicates). Dashed lines indicate 1 standard error above and below the predicted line. (b) Relationship between copulation duration and the number of nymphs produced by pairs which successfully had nymphs, for each of the four treatments in Experiment 1. A linear regression for all treatments combined is shown (dashed line; *N* = 207).

Copulations involving large females lasted much longer (257.8 min ± 9.5 min) than those with small females (208.9 min ± 9.6 min; *F*_1,339_ = 13.24, *p* < .001; Figure 2b). Male body size had no influence on copulation duration (*F*_1,339_ = 0.26, *p* = .607), nor was there an interaction between male and female body size (interaction: *F*_1,339_ = 0.76, *p* = .384). Copulation duration was the main driver for whether pairs produced nymphs or not, with longer copulations being more likely to result in offspring production, and the effect of female size was lost when copulation duration was included in the model (copulation duration: *F*_1,338_ = 169.93, *p* < .001; female size: *F*_1,338_ = 0.26, *p* = .610; Figure 3a). The mean copulation duration that resulted in offspring production was 303.3 min ± 9.0 min, whereas the mean duration for pairs which experienced mating failure was 124.5 min ± 5.8 min (reinforcing the fact that ‘failed matings’ are still non‐trivial in terms of time investment). For pairs which had offspring, longer copulations resulted in more nymphs (*F*_1,202_ = 12.51, *p* = .0005; Figure 3b), and this was also impacted somewhat by male size (*F*_1,202_ = 3.86, *p* = .051) with larger males more likely to have more nymphs (mean = 49.1 ± 2.5) than small males (mean = 41.3 ± 2.9). There was no impact of female size (*F*_1,202_ = 1.06, *p* = .305). However, there was an interaction between male and female size (interaction: *F*_1,202_ = 6.93, *p* = .009) as large males sired more nymphs (mean = 54.2 ± 3.3) from large females than small males (mean = 35.6 ± 5.5), but there was no difference in the number of nymphs sired between large and small males when the female was small (large male mean no. nymphs = 40.6 ± 3.3; small male mean no. nymphs = 44.3 ± 3.1; Figure 2a).

### 3.2 Experiment 2: how mating failure varies over 1–24 h

**FIGURE 4:**
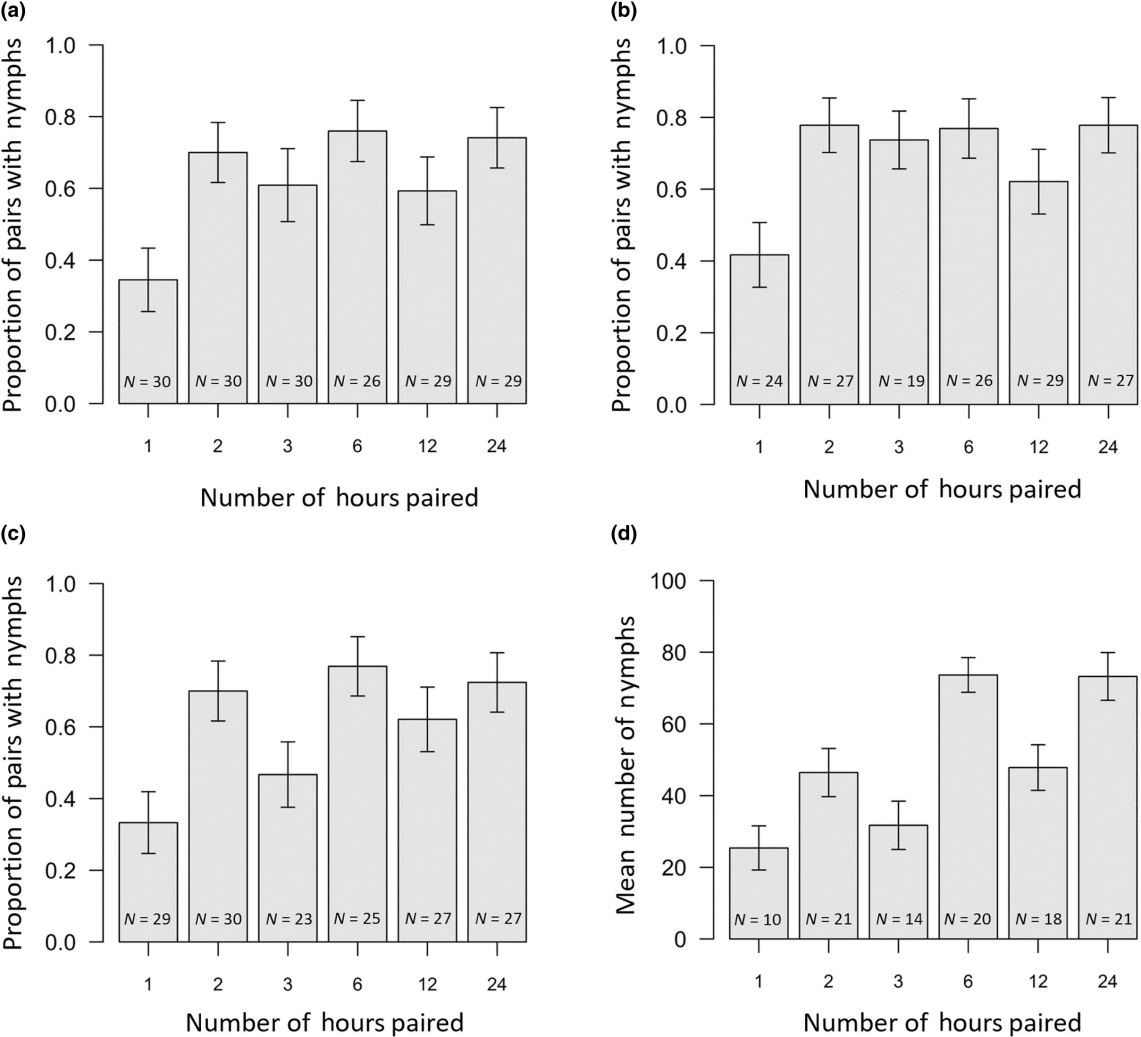
The proportion of pairs which had nymphs in Experiment 2, in relation to the number of hours that pairs were housed together, considering (a) all pairs, (b) only pairs which laid eggs, (c) only pairs in which the female was still alive after she had been left to lay eggs for 7 days. (d) The mean number of nymphs produced by pairs which had nymphs, with respect to treatment. Error bars are standard errors. Sample sizes are given on each bar.

The overall mating failure rate was 40.2%, and the length of time pairs were housed together influenced the mating failure rate (*χ* ^2^_1_ = 4.56, *p* = .033; Figure 4a). In general, the longer pairs were housed together, the more likely they were to produce nymphs. However, mating failure was higher in the 3 h treatment than expected, though this might be because a lot of these females (36.7%) did not lay any eggs, potentially because a number of these females had died before the 7 days of egg laying was finished (23.3%). Therefore, we also tested to see if this relationship held true when (i) excluding all pairs that did not lay eggs and (ii) excluding all pairs in which the female died before the 7 days laying window was finished. When excluding pairs that laid no eggs, there was no effect of pairing duration on mating failure outcome (*χ* ^2^_1_ = 1.35, *p* = .245). However, we note that from Figure 4b it can be seen that fewer pairs in the 1 h treatment had nymphs than pairs in other treatments. The relationship was also non‐significant when pairs with dead females were excluded, but there was the tendency for longer pairing durations to result in lower mating failure rates (*χ* ^2^_1_ = 3.02, *p* = .082; Figure 4c). When considering only pairs which had nymphs, pairs housed together for longer produced a higher number of nymphs (*F*_1,102_ = 15.14, *p* < .001; Figure 4d).

### 3.3 Experiment 3: how mating failure varies over 1–5 days

**FIGURE 5:**
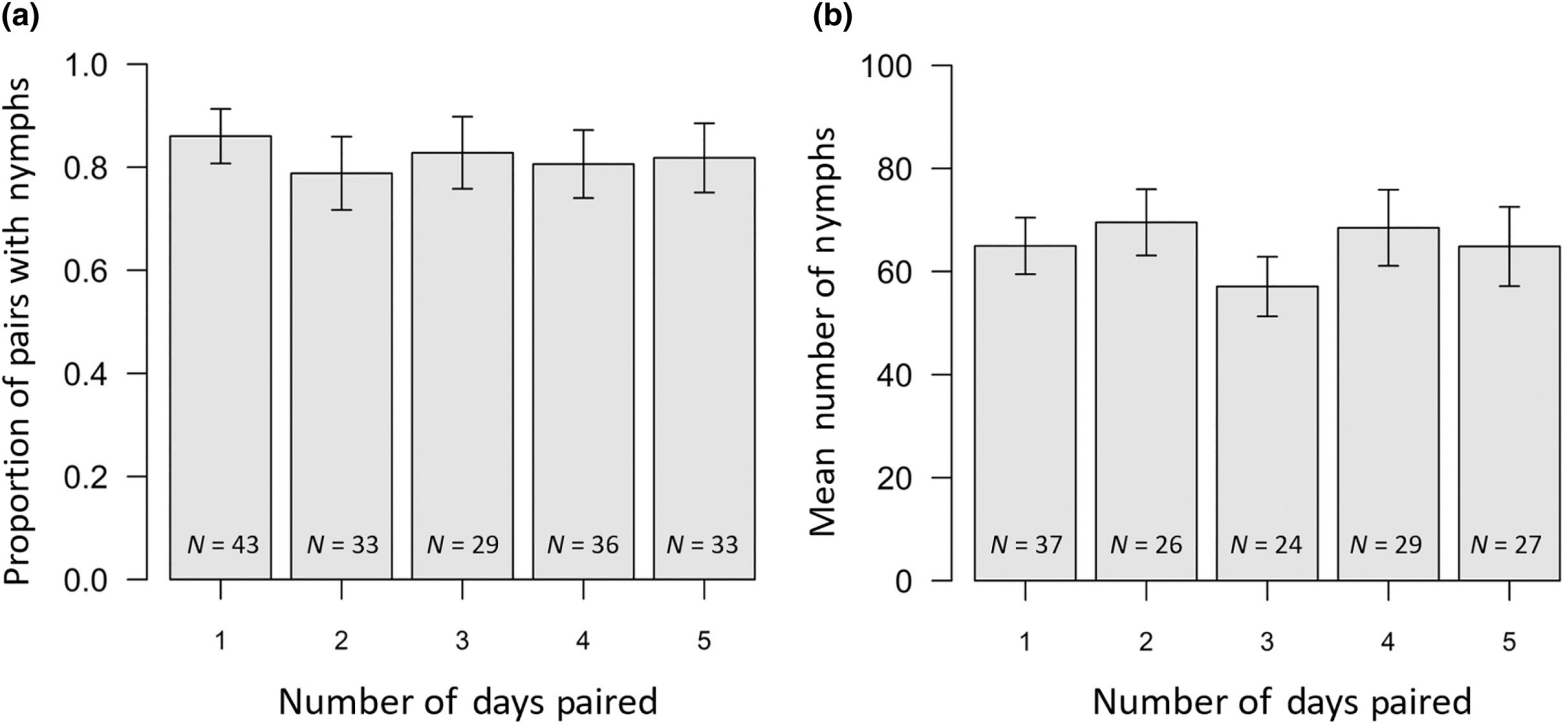
(a) The proportion of pairs which had nymphs and (b) the mean number of nymphs produced by pairs which had nymphs, with respect to the number of days that pairs were housed together in Experiment 3. Error bars are standard errors. Sample sizes are given on each bar.

The overall mating failure rate was 17.8%, which is lower than has been previously reported for experiments during which pairs were paired up for shorter durations. Indeed, being housed together for 24 h seemed sufficient to reduce the extent of mating failure, and the extent of mating failure did not further decrease as the duration of time pairs were housed together increased (*χ* ^2^_1_ = 0.17, *p* = .681; Figure 5a). Nonetheless, we note that approximately one in five pairs still failed to produce offspring. In addition, the number of nymphs sired did not increase with the number of days that pairs were paired up, when considering only those pairs which did not experience mating failure (*F*_1,141_ < 0.01, *p* = .976; Figure 5b). Only one to three pairs in each treatment failed to lay any eggs, therefore we kept all pairs in the analysis when comparing rates of mating failure between treatments.

## 4 DISCUSSION

Frequent occurrences of mating failure are not expected as we would expect primary sexual function to be under strong natural selection (Greenway et al., 2015; Rhainds, 2010, 2019). Indeed, we would also expect some forms of sexual selection to favour individuals able to efficiently supply or receive gametes. Instead, high levels of mating failure suggest some form of post‐copulatory sexual selection, driven by either cryptic female or cryptic male choice (Aumont & Shuker, 2018; Eberhard, 1996). Our experimental manipulation of male and female body size shows clear evidence that mating failure is associated with female body size, with larger females more likely to receive sperm than smaller females. This strongly suggests cryptic male choice on female body size and is consistent with earlier findings in *Lygaeus simulans* (Dougherty & Shuker, 2016; Balfour et al., 2020; see also Gage, 1998). Mating failure was not related to male body size. Furthermore, when we gave pairs more opportunity to mate, in terms of hours or days, we saw a reduction in the levels of mating failure, suggesting that mechanical failure, male or female infertility, or other incompatibilities between specific female and male pairs are not driving mating failure.

As mating failure does appear to be predominantly due to insemination failure (>90% of females which experienced mating failure have no sperm in their spermatheca: Greenway et al., 2017), it seems that males are choosing not to inseminate smaller, potentially less fecund, females. We cannot conclude this definitively from the present data, as perhaps larger females are just more willing to accept male sperm, or put another way, smaller females are either less willing or choosier about the males they accept sperm from (although not in terms of male size). This would be a form of cryptic female choice (Eberhard, 1996) and we cannot exclude it. However, altering copulation duration with regard to female phenotype is a form of CMC, and we do see that pattern here. Of course, this assumes that males have some degree of control of copulation duration in this species, which is likely to be the case as males can be encouraged to disengage from females with gentle touches from a fine paintbrush. Therefore, there is certainly strong circumstantial evidence for CMC. It is also important to note though that size might reflect, or correlate with, a whole range of traits. Therefore, as is typically the case in sexual selection studies, while we can identify non‐random mating outcomes in terms of female size, the exact target of male choice might not be size itself.

Mechanistically, whether mating failure occurs is strongly associated with copulations involving larger females lasting for longer, and longer copulations being likewise less likely to result in mating failure (see also Balfour, Corliss, et al., 2024; Balfour et al., 2020; Dougherty & Shuker, 2014, 2016; Greenway & Shuker, 2015; Micholitsch et al., 2000). For copulations greater than 5 h, the mating failure rate tends to fall below 20%, but then levels off and does not obviously decline any more as copulation duration increases (at least for experiments where bugs are continuously observed for 6 h: see Figure 3a and Balfour, Corliss, et al., 2024; Balfour et al., 2020). Interestingly, this level of mating failure is similar to that seen in Experiment 3, when pairs have up to 5 days to successfully pass and receive sperm. Some level of male and/or female choosiness likely does remain.

Beyond that, it is still unclear what is happening during copulation, and when and how males are assessing females. Clearly some pre‐copulatory assessment is happening (see below; note we have bundled attempted pairings less than 30 min in duration along with no mating attempts in terms of pre‐copulatory choice), but mating failure of course happens once females have been accepted by males (and vice versa), so presumably further assessment is happening. Given that we are focusing on copulation events longer than 30 min, assessment relevant to the final decision of whether to ejaculate looks to be happening during and after deployment of the complex male genitalia (Dougherty et al., 2015; Dougherty & Shuker, 2016; Gschwentner & Tadler, 2000).

We do not know for certain if sperm transfer is continuous in this species. We know that sperm transfer does not start immediately (see Section 2) and that the very long copulations (longer than allowed here, including in excess of 12 h) in the sister species *Lygaeus equestris* seem to be associated with contact mate‐guarding, rather than insemination occurring continually over very many hours (Sillén‐Tullberg, 1981; Shuker et al., 2006; see also Burdfield‐Steel & Shuker, 2014). However, evidence points towards sperm being passed continuously for at least some component of the copulation, since for copulations which do not result in mating failure, longer copulations usually lead to a greater number of offspring sired or, when females are double‐mated, the male which copulates for longer gains a greater share of the paternity (Balfour, Armand, et al., 2024; Balfour et al., 2020). Therefore, longer copulations could be a means for males to transfer a greater amount of sperm to preferred females, another form of CMC.

Another reason for longer copulations with larger females could be that males require more time to navigate the female reproductive tract of these larger females in the first place. However, if this is the case, then it seems once again unlikely that mechanical failure is a primary cause for mating failure, for if larger females have reproductive tracts that are more difficult to navigate, then we would expect to see an increase in the chance of mating failure with these larger females, which is of course the opposite of what we see. Moreover, longer copulations could be a form of mate guarding (Alcock, 1994; Sillén‐Tullberg, 1981), whereby males choose to remain *in copula* with females to prevent other males copulating with them before oviposition occurs. By remaining *in copula* with a female, a male may increase his chances of retaining all of the paternity of that female's next clutch (assuming the female has not already mated previously). In this regard, choosing to copulate with larger females for longer would again be a form of CMC.

Alongside the potential for cryptic male choice, there was also clear pre‐copulatory selection for female body length, with pairings involving large females more likely to result in copulation occurring. This matches our previous work on *L. simulans* (Balfour et al., 2020) and its sister species *L. equestris* (Dougherty & Shuker, 2014). It is expected that males should show a preference for larger females in insects as larger females tend to be more fecund and have a greater potential to lay more eggs and hence produce more offspring (e.g. *Gerris* water striders Fairbairn, 1988; *Streblote panda* moths: Calvo & Molina, 2005; for a more general review on insects see Honěk, 1993). On the other hand, there did not appear to be any pre‐copulatory selection for male body length (something which is sometimes found in this species, just not consistently so: Dougherty et al., 2015; Greenway et al., 2017).

Cryptic male choice may still seem surprising, given that we tend to expect males to be less choosy than females, especially once having committed to copulating, but so too perhaps is (cryptic) mating failure. After all, why would males choose to not transfer sperm after having already gone through the time, energy, and resource costs for finding, obtaining, and engaging in copulation with a female? Surely it would be more costly to the male to not transfer sperm at this point? However, perhaps sperm is a more costly resource to produce than is typically thought. Traditionally, sperm was seen as a cheap and easy resource to produce, as opposed to eggs which were seen as more difficult and costly to produce (Bateman, 1948; Hayward & Gillooly, 2011; Parker, 1982). However, we now know that males can become sperm depleted (e.g. in mammals: Preston et al., 2001; birds: Pizzari et al., 2003; amphibians: Hettyey et al., 2009; fish: Smith et al., 2009; insects: Damiens & Boivin, 2006), and therefore, sperm may not be as cheap and easy to produce as once thought. Given the widespread occurrence of strategic sperm allocation by males in the context of sperm competition (Kelly & Jennions, 2011; Simmons, 2001; Wedell et al., 2002), we should perhaps be prepared to find more examples of CMC if we look for it.

CMC is also surprising in another context, namely that it begs the question as to why males do not, or are not able to, assess females adequately before fully engaging in copulation. One answer is that maybe males are not fully able to assess females until genitalic contact is complete or require further information gained during the preliminaries of copulation. This would seem the most likely alternative to us. However, maybe copulations without sperm transfer are not about mating at all but instead serve some other function. Whatever that function is, it would have to overcome the time and energy costs of being *in copula*, which include reduced mobility. One idea we have entertained is that being an aposematic species might mean that being in tandem might send out a bigger ‘we are toxic’ signal to potential predators, compared to a solitary individual with their smaller ‘I am toxic’ signal (this idea builds on one of the accepted explanations for the aggregations found in aposematic species, including insects: see Ruxton et al., 2018). However, we suspect that any effect like this would do more to perhaps mitigate the costs of mating (including costs via predation) rather than select for the ungainly back‐to‐back position for non‐mating purposes. Moreover, if males and females get together for non‐copulatory purposes, we are not sure that we would predict the relationship between time spent together and mating failure, as the non‐copulatory tandem pairs should presumably come together and persist for reasons independently of how long it takes sperm to be transferred in the copulating pairs. As such, we are not sure that there is currently a good argument for pairs forming for non‐sexual reasons in *L. simulans*, but we acknowledge that it remains a possibility.

In conclusion, we have brought together evidence to argue that CMC is a driver of mating failure in *L. simulans*. If we are right, then this not only adds to the evidence for CMC in animals but to the growing body of literature on male mate choice more generally. Our study is by no means conclusive as we have not yet been able to definitively tease apart male and female effects. There remains the possibility that larger females just have a higher desire to receive sperm than smaller females. Therefore, we need to develop a solid method of disentangling male and female effects. One way in which this might be done would be to alter the treatment of males, blind to the females, and see if we change the value of a given female to a male, and definitively show the male role. This is something we are currently working towards.

## AUTHOR CONTRIBUTIONS

**Vicki L. Balfour:** Conceptualization (equal); data curation (equal); formal analysis (lead); investigation (equal); methodology (lead); project administration (equal); writing – original draft (lead); writing – review and editing (equal). **Mélissa Armand:** Data curation (equal); writing – original draft (supporting). **David M. Shuker:** Conceptualization (equal); funding acquisition (lead); methodology (supporting); project administration (equal); resources (lead); supervision (lead); writing – original draft (equal); writing – review and editing (equal).

## CONFLICT OF INTEREST STATEMENT

The authors declare no competing nor conflicting interests.

## Data Availability

The research data underpinning this publication can be accessed at https://doi.org/10.17630/d7ce6517‐b2b6‐49b1‐81c5‐1e99057fe45b.
